# Analysis of Flood Evacuation Process in Vulnerable Community with Mutual Aid Mechanism: An Agent-Based Simulation Framework

**DOI:** 10.3390/ijerph17020560

**Published:** 2020-01-15

**Authors:** Zhiqiang Wang, Jing Huang, Huimin Wang, Jinle Kang, Weiwei Cao

**Affiliations:** 1 State Key Laboratory of Hydrology-Water Resources and Hydraulic Engineering, Hohai University, Nanjing 210098, China; 2 Institute of Management Science, Business School, Hohai University, Nanjing 211100, China

**Keywords:** flood evacuation, vulnerable community, mutual aid mechanism, agent-based model, simulation

## Abstract

Timely and secure evacuation of residents during flood disasters or other emergency events is an important issue in urban community flood risk management, especially in vulnerable communities. An agent-based modeling framework was proposed in order to indicate how the community properties (e.g., community density and percentage of vulnerable residents), residents’ psychological attributes (e.g., flood risk tolerance threshold) and mutual aid mechanism affect the flood evacuation process. Results indicated that: (1) The community density negatively affected the flood evacuation efficiency. The greater the density of the community, the longer the evacuation time. (2) There was a negative correlation between the flood risk tolerance threshold of residents and evacuation efficiency. (3) The proportion of vulnerable resident agents had opposite effects on the evacuation efficiency of different types of communities, which was to negatively affect low-density communities and positively affect high-density communities. (4) Mutual aid mechanism can reduce evacuation time in low-density communities, and the effect was more pronounced with a higher proportion of vulnerable resident agents in the community. These findings can help managers to develop better emergency evacuation management for urban communities.

## 1. Introduction

Flood disasters are a major threat to human society and economic systems, and they can easily wipe out the wealth accumulated in the past [1]. In recent years, the frequency and scope of flood disasters have increased significantly as well as the economic losses and human casualties caused by floods (https://www.cred.be). Actually, casualties and economic losses can be reduced or prevented if people arrive shelters and transfer the assets before the flood disasters. Therefore, timely and securely evacuation of residents is of great importance during flood disaster events [2]. Nowadays, with the acceleration of urbanization in China, people gather together in the form of communities. Therefore, emergency evacuation of urban communities is becoming more important and serious. Vulnerability theory has been widely applied in the research of natural disasters. Vulnerability is generally considered as the possibility that an individual or a group is exposed to and affected by natural disasters [3,4,5]. Individuals or groups have different vulnerabilities due to their own characteristics, such as exposure, coping ability, and adaptability. When faced with natural disasters, residents can be divided into vulnerable residents and non-vulnerable residents according to their ability to resist natural disasters. The vulnerable residents refer to those individuals or groups with weak ability to resist disasters, such as the disabled, the elderly, children, etc. The proportion of vulnerable residents can be used to show the vulnerability of a community. The higher the proportion of vulnerable residents, the more vulnerable the community. In highly vulnerable communities (communities with many elderly and children), due to slow disaster response, insufficient response capacity and resources, the emergency evacuation of vulnerable communities is more prominent and severe. To mitigate the adverse effects of flood disasters on urban residents, better evacuation planning based on the results of emergency evacuation studies is becoming critical in flood risk management. Due to this, the study of community flood emergency evacuation has attracted more and more attention in recent years.

In general, emergency evacuation is considered as a traditional routing problem and many improved classical algorithms have been proposed to solve this problem [6,7]. In fact, the emergency evacuation is a complex problem and is affected by many factors such as psychology [8], demographics [9], human relations [10], risk warning [11,12], and so on. Therefore, at present, many studies recognize the emergency evacuation problem from the perspective of complex system and use computer models to simulate the evacuation process [13,14]. There is no doubt that the purpose of emergency evacuation is to evacuate people to the safe areas as quickly as possible. Therefore, evacuation time, evacuation distance, evacuation ratio and other indicators are used to evaluate the efficiency and capability of emergency evacuation, and these indicators are also the most concerned by decision-makers. As for the simulation models, in the existing studies, agent-based model (ABM) has been suggested as an appropriate tool to solve the kind of complex system problems such as emergency evacuation. ABM is a type of computational model that can provide the most natural description and simulation of complex adaptive systems, and implement the assumptions of model [15]. Through the simulation of the behaviors and interaction of autonomous agents in the ABM, the dynamic feedback of subsystem components, their inherent complexities and their effects on the system can be captured. The applications of ABM range across virtually all kinds of disaster evacuation problems and well beyond the usual ones for simulation [16]. For example, Zou et al. carried out an agent-based modeling of people from trains and platforms in a typical subway station. Parametric investigations found out the impacts of some key parameters on evacuation time and provided some valuable insights for understanding the potential causes of delay of evacuations [17]. D’Orazio et al. presented an agent-based model to describe phases and rules of motion for pedestrians and found that evacuation paths choice depending on configuration of environment and damage distribution after an earthquake [18]. Liang et al. developed a two-level regional disaster evacuation model by coupling two agent-based models to simulate hurricane evacuation traffic in New Orleans and confirmed that the proposed model performs well in terms of high model accuracy [19]. Mostafizi et al. proposed an agent-based modeling framework to evaluate vertical evacuation behavior and shelter locations for a near-field tsunami hazard and revealed that the non-linear correlation between the aforementioned characteristics of the vertical evacuation shelter on the expected mortality rate [20]. Wijerathne et al. presented an HPC enhanced agent-based model developed with the aim of quantitatively estimating the strategies for accelerating emergency mass evacuations, like tsunami evacuation. It is demonstrated that the system has high strong scalability up to 10 million agents was simulated [21]. Liu et al. proposed a shelter assignment and routing strategy for evacuating households at the 2011 Brisbane flood event and the results showed that communities located in the east and west of Brisbane are scarcely covered by existing shelters [22]. These simulations showed the whole procedure of an emergency evacuation, found out a number of evacuation problems and provided possible improvements for emergency response.

Although many studies have been conducted, most studies on emergency evacuation were restricted to single building [23,24] or large-scale evacuations [22,25], while few studies focused on community-scale evacuation [6]. In addition, in the existing study of emergency evacuation of urban communities, there are two important issues rarely considered by researchers. The first one is that communities in China’s small and medium-sized cities are becoming increasingly vulnerable. It should be noted that in China, with the rapid economic and urbanization development, young labor force prefers to work in big cities, resulting in the phenomenon that some communities in small and medium-sized cities are dominated by the elderly and children. That means this kind of communities are at an increasing risk of flooding in terms of lower emergency response ability. Emergency evacuation in vulnerable communities under flood disaster is becoming a more critical issue. Therefore, it is important and necessary to pay attention to the emergency evacuation of vulnerable communities in order to quickly and orderly evacuate vulnerable residents. The second one is the mutual aid behaviors among residents in the communities during flood emergency evacuation. According to the results of our several field investigations and questionnaire surveys in Jingdezhen, Jiangxi Province, China, it was found that during flood emergency evacuation, residents generally had the consciousness and behaviors of helping each other and going to shelter together [1]. Moreover, the behaviors of helping each other to evacuate to the shelter together can be motivated, encouraged by the local government. In this paper, the act of helping each other escape the effects of flood disasters was defined as mutual aid behaviors. Accordingly, encouraging and motivating residents to produce such behavior was defined as a mutual aid mechanism, and the proportion of non-vulnerable residents who are willing to help was used to show the strength of the mutual aid mechanism. Through analyzing and understanding the role of residents’ mutual aid mechanism in emergency evacuation can help local decision-makers to perfect reasonable emergency evacuation plans. However, the relationship between mutual aid behavior and emergency evacuation efficiency has not received much attention in existing studies.

Considering the limitation of the existing studies and motivated by the two important issues mentioned above, this study presented an agent-based modeling framework to simulate the flood evacuation in vulnerable communities and this framework incorporated the resident psychological model and transportation network model. The aims of this study were to find out how the community properties (e.g., community density and percentage of vulnerable residents), resident’s psychological attributes (e.g., flood risk tolerance threshold) [26,27] and mutual aid mechanism affect the emergency evacuation efficiency, summary the characteristics of emergency evacuation in vulnerable communities, and give corresponding improvement suggestions of better response to emergency evacuation. Specifically, the simulation results of this study can easily present spatial analysis and statistics results, such as evacuation time, average evacuation time, number of agents on the road, the congestion of the road, etc. Through analyzing the evacuation simulation results, this study can help decision-makers at the community level to better understand the dynamic characteristics of community evacuation behavior and make effective emergency evacuation plans. It is of great practical significance for community emergency evacuation and flood risk management. A case study of synthetic community was conducted to demonstrate the feasibility and applicability of the framework.

## 2. Methodology

### 2.1. Framework of Agent-Based Model

This evacuation process simulation mainly involved two components: agents and transportation network. Thus, the proposed agent-based model took both human components (community residents and staff, and their decision-making process) and evacuation transportation network into consideration.

**Figure 1:**
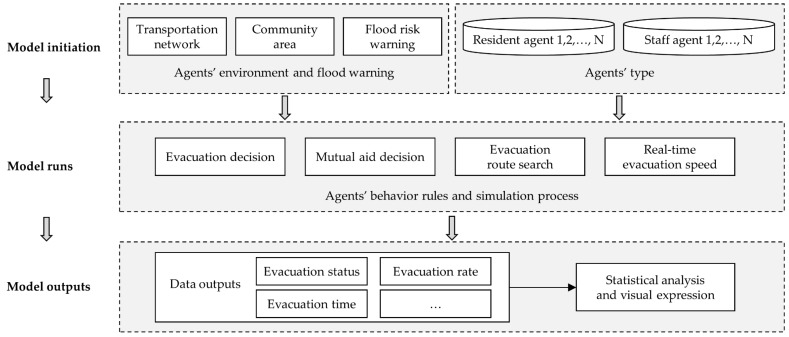
Framework of the agent-based model for flood evacuation process simulation. Each time step, the flood risk perception of agents was updated and agents took action.

The agent-based model framework was structured in three steps: model initiation, model runs, and model outputs (as shown in Figure 1). In the model initialization step, the agents’ environment and the agents were created, and the flood risk warning was also issued. When the agent-based model running, evacuation decision-making, mutual aid decision-making, evacuation route searching, and other behavior rules were running to finish the simulation process. With regard to the model output step, some data results at system level were outputted, analyzed and visualized. The focus of the study was to investigate the impact of various factors on the flood evacuation efficiency (such as evacuation status, percentage of evacuated agents and evacuation time, etc.). Therefore, in the model statistical analysis step, some main issues were considered as below:

- The impacts of the community density on the evacuation process
- The impacts of the average flood risk tolerance threshold on the evacuation process
- The impacts of the percent of the vulnerable resident agents on the evacuation process
- The impacts of the community mutual aid mechanism on the evacuation process

### 2.2. Construction of the Agent-Based Model

#### 2.2.1. Flood Risk Warning

In the mainland of China, the city local meteorological authority is responsible for the release of early flood risk warning signals within its administrative area. The flood risk warning signal is divided into four levels, which are represented by blue warning, yellow warning, orange warning and red warning. And the red flood risk warning signal is the highest level. When the red flood risk warning signal is issued, according to disaster management requirements, the community residents committee staff are required to inform and assist residents to evacuate, especially the vulnerable individuals.

In this study, let $FRW_{t}$ denote the value of flood risk warning from authority department at time step t (i.e., $FRW_{t}\in [0,1]$). The higher the value of $FRW_{t}$, the greater the flood risk. When the value of $FRW_{t}$ is set to 1, it means the highest flood risk warning, and all the resident agents and community residents committee staff agents must evacuate to the shelter eventually. Since the authority department, in reality, broadcasts the flood risk warning information to all the residents and staff in its administrative area, all agents will receive the same flood risk warning information at each time step [26].

#### 2.2.2. Resident Agents and Behavior Rules

An agent is defined by the attributes and behavior rules. Every agent’s response to the environment or other agents is based on its own attributes and behavior rules. The following sections introduced how to define the resident agent’s attributes and decision rules.

##### Resident Agent Attributes

There are various variables of resident that can affect the real evacuation process. It is difficult and challenging to completely include all of impact factors in one model when the empirical evacuation data is lacking. Therefore, in this study, only important factors of residents were taken into account [28]. The agents’ attributes were classified as physical attributes that were related to its evacuation process, and psychological attributes that were related to its response to flood warnings (as shown in Table 1).

In this study, the resident agents were divided into two categories: non-vulnerable resident agents and vulnerable resident agents (as shown in Table 2). The percentage of the vulnerable resident agents represented the extent of the vulnerability of the community. The moving speed of non-vulnerable resident was faster than vulnerable resident. Besides, the non-vulnerable resident agent can response to the flood event by themselves while the vulnerable resident only evacuates when the community staff inform them or other non-vulnerable resident help them to evacuate.

##### Resident Agents’ Flood Risk Perception

In this study, the flood risk perception of resident agents (denoted by a continuous variable $FRP_{i}$, $FRP_{i}\in [0,1]$) referred to their feeling of the extent of flood risk in the community where they stay, which would affect their evacuation decision. In each time step, every resident agent’s flood risk perception was calculated and updated. If one resident agent’s flood risk perception exceeds its specified flood risk tolerance threshold, the resident agent would consider taking action to evacuate to the shelter. However, the flood risk perception can be affected by many factors, such as past flood experience, neighbors’ behavior and new information received [1,29,30].

In this study, the calculation of flood risk perception was based on factors identified through the literature review. When the community was hit by a flood event, the resident agent i mainly obtained flood risk information from the flood risk warning $I_{i,t}^{W}$ and neighbors’ flood risk perception $I_{i,t}^{N}$ (as shown in Equations (1) and (2), respectively). Some studies have also considered the impact of social media on flood risk perception [26,31]. However, this study argued that in small communities, the impact of government warning and neighbor behavior is far greater than that of social media.

(1) $$I_{i,t}^{W}=FRW_{t}$$

(2) $$I_{i,t}^{N}=\frac{\sum_{j=1}^{n}c_{i,j}FRP_{j,t}}{\sum_{j=1}^{n}c_{i,j}}$$

where $FRW_{t}$ denotes the flood risk waring at time step t, $FRP_{j,t}$ denotes the flood risk perception of agent j, and if agent j and agent i are in the same community grid, the $c_{i,j}$ takes 1, otherwise $c_{i,j}$ takes 0. In other words, the $I_{i,t}^{N}$ is the average value of flood risk perception of resident agents in the same community grid.

Many previous studies using a set of weighting factors to formulate agents’ flood risk perception driven by multiple information sources. In this study, the approach was adopted and two main information influence parameters $\alpha_{i}$ and $\beta_{i}$ were set to represent the influence of flood risk warning and neighbors’ flood risk perception for agent i, respectively. Thus, for agent i, the new flood risk information obtained from multiple sources can be represented by Equation (3).

(3) $$\Delta I_{i,t}=\alpha_{i}I_{i,t}^{W}+\beta_{i}I_{i,t}^{N}$$

When new information on flood risk was obtained, the agent i would update its flood risk perception. However, because of past experience, information reliability, and information comprehensibility, different agents had different levels of trust in the new information. In this study, for agent i, a weight parameter $\omega_{i}$ was set to represent the extent of its willing to accept the new information it obtained at time step t. Therefore, the flood risk perception of agent i at time step t can be represented by Equation (4).

(4) $$FRP_{i,t}=FRP_{i,t-1}+\omega_{i}\times \Delta I_{i,t}$$

So far, the process of how agents update their flood risk perception was modeled.

##### Resident Agents’ Evacuation Decision

At time step t, the agent would make an evacuation decision (denoted by a binary variable $ED_{i,t},ED_{i,t}\in \{0,1\}$) to evacuate or not when the flood risk warning was issued (as shown in Equation (5)). In this study, if one agent’s flood risk perception $FRP_{i,t}$ larger than its flood risk tolerance threshold $RT_{i,t}$ at time step t or the agent already on the way to the shelter in the last time step t−1, the agent would decide to evacuate or continue to evacuate to the shelter. In other cases, the agent would stay at home.

(5) $$ED_{i,t}=\left\{ \begin{array}{ccc} 0 & if & FRP_{i,t}<RT_{i,t}\\ 1 & if & FRP_{i,t}>RT_{i,t}\;\mathrm{or}\;ED_{i,t-1}=1 \end{array}\right.$$

##### Resident Agents’ Mutual Aid Process

For the non-vulnerable resident agents who willing to aid, if the mutual aid mechanism was on and they had decided to evacuate, they would check whether there were vulnerable resident agents in their same community grid. If there was at least one vulnerable resident agent did not evacuate, the non-vulnerable resident agents would move to their nearest vulnerable resident agent’s location and form one-on-one evacuation team. Then, they would move to the nearest road edge first and go to the shelter together through taking the shortest route. If there was no vulnerable resident agent in their community grid, the non-vulnerable resident agents would go to the shelter directly. For the non-vulnerable resident agents who did not want to afford help, they would go to the shelter directly regardless of the mutual aid mechanism was on or not. When the resident agents arrived at the shelter, they would change their evacuation status to ‘arrived shelter successfully’.

**Figure 2:**
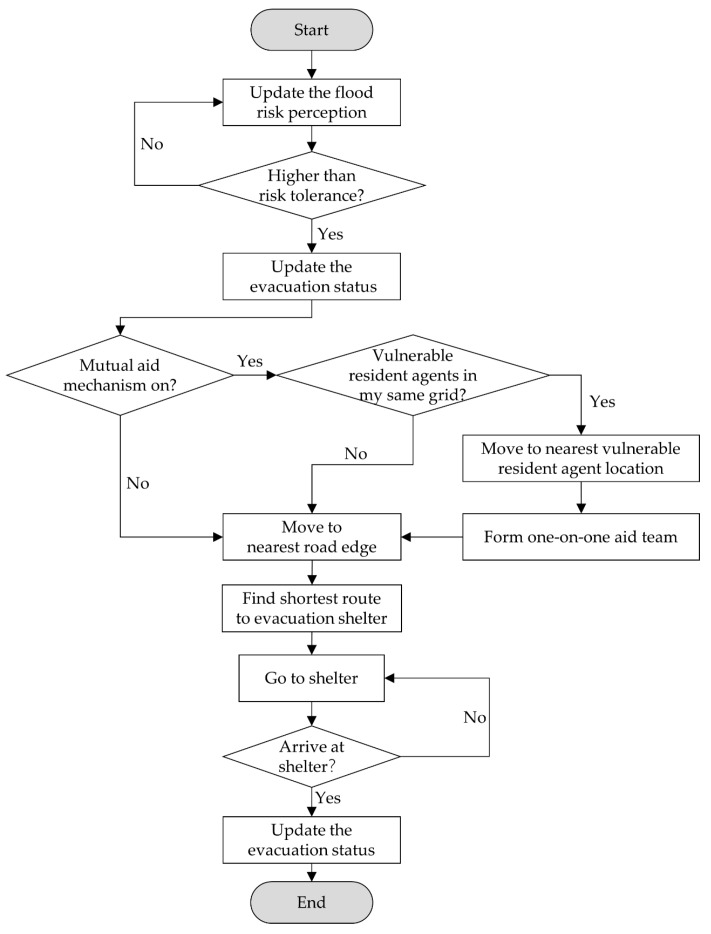
The decision-making process of resident agents.

So far, the resident agents’ attributes and its behavior rules has been constructed. The simulation process end when the tick time agreed with the time set or all the resident agents arrived at the shelter. Figure 2 presented a completed decision-making process flowchart of the resident agents.

#### 2.2.3. Community Residents Committee Staff Agents and Behavior Rules

The staff agents represented the community residents committee staff in the real world. In each community grid, there were certain number of staff to responsible for the daily work in China. In the face of the flood risk, the community residents committee staff should inform and assist the residents to evacuate if the flood risk alert is red warning. In this study, the staff agents need to response to flood event and help the vulnerable resident agents to evacuate to the shelter. In this study, the attributes of the staff agents were the identification number, community grid number and evacuation status.

**Figure 3:**
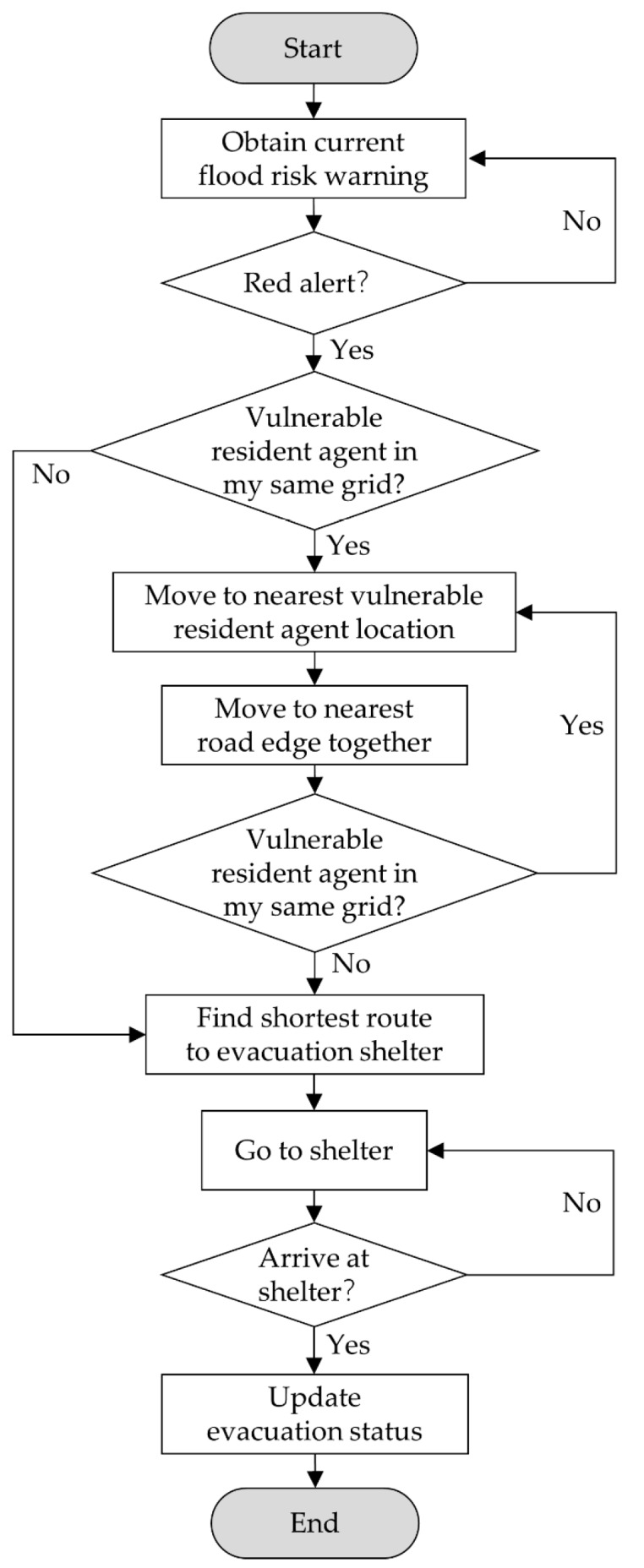
The decision-making process of community residents committee staff agents.

Figure 3 showed the completed flowchart of the decision-making process of the staff agents during the flood event. The staff agent firstly checked whether the flood risk warning was red alert, if yes, the staff agent would find the vulnerable resident agents in its same community grid and move to the nearest vulnerable resident agent location. Then, the staff and the vulnerable resident agent would go to the nearest road edge together. If there were no more vulnerable resident agents in its community grid, the staff agent and the vulnerable resident agent just found would go to the shelter directly. Otherwise, the staff would ask the vulnerable resident it found last time to go to the shelter alone by the shortest route, and the staff would go to find the next vulnerable resident in its community grid. After repeating the searching process several times, there were no more vulnerable resident agents, the staff agent would move to the shelter at last. All agents who arrived the shelter would update the evacuation status to “arrived shelter successfully”.

#### 2.2.4. Road Network and Traffic Rules

The road network plays a key role in emergency evacuation and emergency management. A road network is a system of interconnecting lines and points (called edges and nodes in network science) that represent a system of streets or roads for a given area, and it describes a structure which permits either vehicular movement or flow of people [32]. Generally, the road network system includes road network, vehicles and people in the network and move rules that regulate the movements and interactions of vehicles and people. Thus, there were two main components in a road network system simulation: (1) the road network itself; and (2) the movement rules of the road network that all vehicles and people should follow. However, it is challenging to explicitly include all road network features in simulation model [28]. Therefore, many studies have suggested using a simplified road network which consists of a number of nodes, edges and edge weights [33,34]. A set of edges and nodes can be routed from one node to another in a road network. In this study, the road network was set to two-way road, and the Dijkstra’s algorithm [35] was applied to find the shortest paths between nodes.

As for the movement rules, it stipulates the movement and interaction mechanisms of the vehicles and people in the road network. Among a variety of transportation network simulation methods, the Nagel–Schreckenberg model (N-S model) proposed by Nagel and Schreckenberg [36] is widely used to simulate the movement of the vehicle and the speed of the vehicle was adjusted by the road maximum limit speed, the speed of ahead vehicle, and a safe distance, etc. While regarding the short-distance evacuation process in a population-intensive community, people mainly evacuate by walking instead of using vehicles. Therefore, people’s evacuation speed is mainly affected by their own walking speed and the density of people on the road. The calculation results of statistical data in many studies showed that the speed of pedestrians is 1–2 m/s, and the pedestrian walking speed would be increased or decreased under special circumstances. With regard to the density of people on the road, it reflects the intensity of people in a certain space for each time step. Specifically, it refers to the value that the number of people on the road unit where you stay plus the number of people on ahead road unit, then divide it by 2. Although there are various models to study the relationship between the evacuation speed and the people density [10,37,38,39], they have similar general speed rules. In this study, the relationship between the speed of agent and agent density was referenced by the SGEM model [40] developed by Wuhan University and City University of Hong Kong (represented by Equation (6)).

(6) $$v_{i}=\left\{ \begin{array}{l} 1.4\\ 0.0412\rho_{i}{}^{2}-0.59\rho_{i}+1.867\\ 0.1 \end{array}\right.\begin{array}{c} if\\ if\\ if \end{array}\begin{array}{c} \rho_{i}\leq 0.75\\ 0.75<\rho_{i}\leq 4.2\\ \rho_{i}>4.2 \end{array}$$

where $v_{i}$ denotes the speed of agent i, $\rho_{i}$ denotes the agents density (agents/road_unit) on the road unit where agent i stay. The speed of all non-vulnerable resident agents and committee staff agents were $v_{i}$, and the speed of vulnerable resident agents was set to 0.8 times of $v_{i}$.

In order to find more detail information during the evacuation process, in this study, the road congestion index (RCI) was used to reflect the degree of crowdedness in the transportation network. It can be estimated by calculating the proportion of agents whose speeds reduce more than a certain percent at each time step (as shown in Equation (7)).

(7) $$RCI=\frac{\sum_{i=1}^{n}SSA_{i}}{n}$$

where RCI denotes the extent of the road congestion, n denotes the total number of resident agents who are evacuating to the shelter, and the $SSA_{i}$ is a binary function which is used to indicate whether the speed of agent i has decreased significantly, if its speed less than 50% of its max moving speed, $SSA_{i}$ takes 1, otherwise $SSA_{i}$ takes 0. In addition, the higher the RCI value, the more congested the road.

#### 2.2.5. Model Assumptions and Outputs

It is very difficult to consider all the impact factors in one evacuation simulation model. Therefore, some main assumptions were set to simplify the model and ensure the availability and credibility of results. They were: all residents in the community were required to carry out emergency evacuation, and all residents were in their homes when the flood risk warning was issued; the evacuees were assumed to have good knowledge about the transportation network and they will head to the nearest road node first and then look for the shortest route to the shelter.

Besides, the community is a complex system due to it consists of residents, residents committee staff, roads, etc. When simulating the process of emergency evacuation, each agent interacted and influenced each other under certain rules. These interactions and influences happened in the process of flood risk perception update, evacuation decision, road congestion and other aspects. Actually, each agent makes different decisions during these interactions, and these behaviors were assumed to occur in a stochastic manner. Hence, the community evacuation system turns into highly randomness, nonlinear and noncontinuous. For such a system, obtaining the closed-form analytical solutions become very difficult.

In similar studies, numerical simulation methods were usually used by researchers to solve such problems [41,42,43]. Monte Carlo methods, which rely on repeated random sampling to obtain numerical results, are often used in physical and mathematical problems [44]. Therefore, in order to obtain stable numerical results, in this study, the Monte Carlo approach was applied to analyze the impacts of different agent variables on the evacuation process. The model was executed 500 times on the netlogo 6.0.4 platform per scenario to ensure the stabilization of numerical results. The 15% trimmed mean method was applied to further reduce the randomness and contingency of numerical results [45]. In addition, in order to improve the efficiency of the model, the model was optimized based on the method proposed by Steven et al. [46].

Multiple indicators were used to measure agents’ evacuation behaviors at the system level (i.e., a community). These indicators were: (1) road congestion index $RCI_{t}$, representing the extent of the road congestion at time step t. (2) resident agents’ evacuation status, where were the percentage of the resident agents with different status (i.e., start to evacuate, on the way to the shelter and successfully evacuated to the shelter) at time step t. (3) total evacuation time, representing the time cost to evacuate all the residents to the evacuation shelter; average evacuation time, representing the average value of all the resident agents’ total evacuation time.

### 2.3. Synthetic Community and Scenario Design

#### 2.3.1. Synthetic Community Design

**Figure 4:**
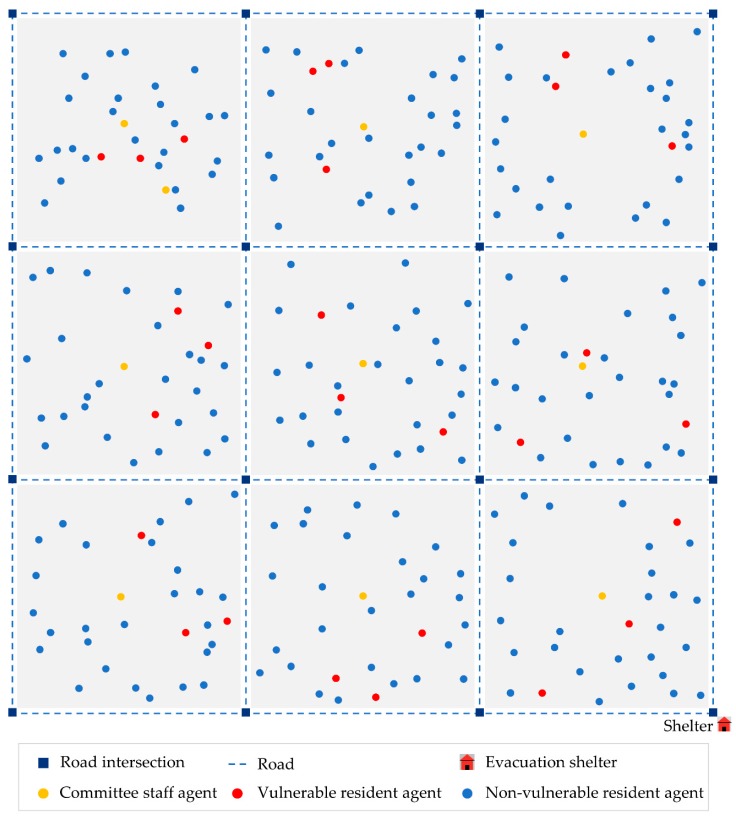
Illustration of the synthetic community. The community consisted of a road network, nine community grids, one shelter and a number of community residents committee staff agents and resident agents.

In this study, the agent-based model proposed above was applied in a synthetic community (as shown in Figure 4), which was constructed according to the characteristics of urban communities’ structure and the mechanism of community grid management in China. The synthetic community included a transportation network, nine community grids and an evacuation shelter in the bottom right. There were sixteen intersection nodes and twenty-four roads in the regular lattice road network, and the length of each road was 100 units. Each community grid contained a number of community residents committee staff and residents, and these community grids were connected by roads. In order to make the simulation more in line with the real situation of the community, all agents were randomly distributed in their corresponding community grid.

In addition, the impacts of the number, distribution, and capacity of evacuation shelter were worth to study, and the results can be useful in the evacuation plan or shelter plan. However, it is difficult to analyze all the factors in one study. Therefore, in this study, only one evacuation shelter was built and was assumed to be able to accommodate all agents.

#### 2.3.2. Scenario Design

Table 3 showed the values of model parameters. Those model parameters were important to build the agent-based model and necessary to format the agents’ status and behaviors. As mentioned in Section 2.2.2, the resident agents obtained information from multiple separate sources and each agent can decide how much it adheres to its past risk perception when new information was available. However, the impact of different weights of information sources and learning rate were not the focus of this study. Therefore, with reference to previous studies, the weight of government information $\alpha_{j}$ and the weight of neighbor behavior $\beta_{j}$ were set to 0.5 and 0.5, respectively, and the learning rate $\omega_{j}$ was sampled from a normal distribution with a mean of 0.5 and standard-deviation of 0.1. In addition, the value of the government flood risk warning FRW was set to 1, which means the red warning of flood risk. Moreover, the agents only received one flood risk warning at the beginning of the model execution, and would not receive any other flood risk warning in the following simulation.

With the synthetic community as a case study area, the aims of this study were to explore how residents’ heterogeneous behaviors (i.e., flood risk tolerance threshold), community properties, and mutual aid mechanism affect the emergency evacuation process. Four different scenarios were designed based on five important parameters (as shown in Table 4). The five parameters were respectively the flood risk tolerance threshold of the resident agents, the community density, the percentage of vulnerable resident agents, the mutual aid mechanism status, and the proportion of the non-vulnerable resident agents who willing to aid.

The first scenario aimed to investigate the impact of the community properties on the evacuation process. There are many variables can be used to measure the community characteristics, such as community size, community density, community layout, education level, etc. In this study, only community density was concerned, as it may significantly affect traffic during an emergency evacuation process. The second scenario investigated how the flood risk tolerance threshold of resident agents affects the evacuation process. The different threshold of flood risk tolerance represents the different residents’ response to flood risk. Appropriate flood risk tolerance threshold could enable more efficient evacuation. The third scenario explored the effects of the community vulnerability (expressed by the percentage of the vulnerable resident agents) on the evacuation process under different community density. In the fourth scenario, the potential influence of mutual aid mechanism on emergency evacuation was investigated which combined with other three influencing factors.

## 3. Results and Discussion

### 3.1. Impacts of Community Density on the Evacuation Process

The first scenario was mainly to investigate the impact of community density on the emergency evacuation process. To be specific, this scenario addressed two questions: (1) Does the community density affect the evacuation process? (2) To what extent does the community density affect the evacuation efficiency? The first question was to demonstrate that the community density can affect the evacuation process and the second question was to attempt to evaluate the importance and the impact of community density in evacuation process.

**Figure 5:**
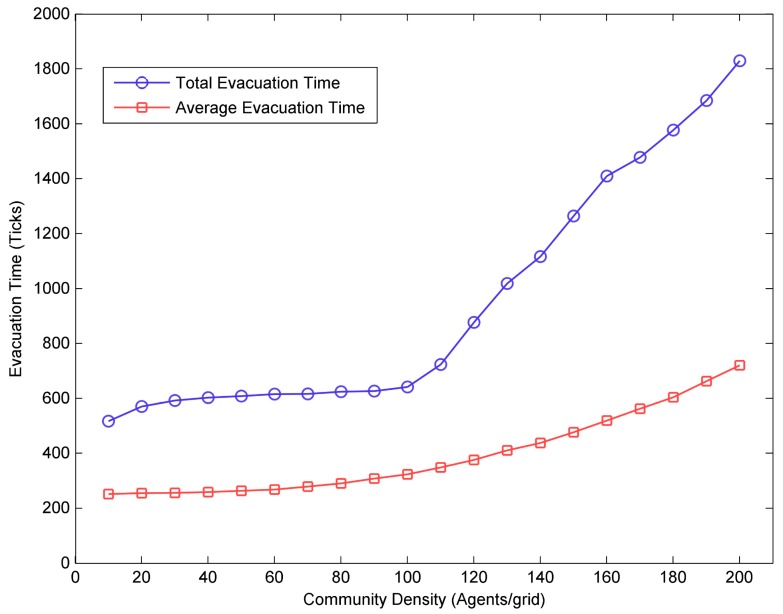
The impacts of community density on the total evacuation time.

Figure 5 showed the simulation results for the first scenario in which the mean value of flood risk tolerance threshold was 0.7, the percent of vulnerable resident agents was 10% and no mutual aid behavior between resident agents. The results indicated that the higher the community density, the longer the evacuation time required. In other words, the community density had a negative correlation with the evacuation efficiency. Moreover, when the community density was less than 120 agents/grid, the total evacuation time and average evacuation time gradually increased with the increase of community density. However, when the community density was greater than 120 agents/grid, the total evacuation time and average evacuation time increased rapidly. These results were similar to the findings in most published studies [26,28,41].

**Figure 6:**
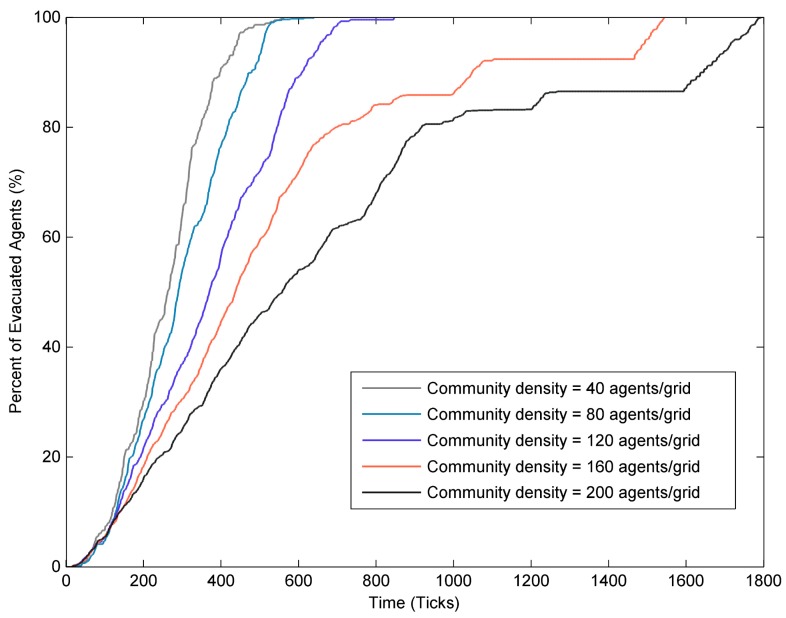
The percent of evacuated agents with time under different community density.

In order to explore how community density affected the evacuation process, the changes in the percent of evacuated agents with time in communities with different densities were investigated (as shown in Figure 6). It was clear that the lower the community density, the faster the percent of the evacuated agents reached 100%. Furthermore, for every increase in community density, more and more evacuation time was needed to reach the same evacuation ratio, especially in the case of high evacuation ratio situation. In other words, when achieving a high level in the evacuation ratio, the increase in evacuation time was much larger than the increase in community density [28]. For example, considering the time needed for 90% of the resident agents evacuated to the shelter, when the community density increased from 40 to 80 agents/grid, the evacuation time increased by 20.65%, while when the community density increased from 160 to 200 agents/grid, the evacuation time needed increased by 57.06%. In both cases, the community density increased by 40, but the increase of the total evacuation time of the latter (high-density community) was far higher than the former (low-density community). These results indicated that achieving high level of evacuation ratio was much more difficult in high-density community than in low-density community due to the marginal evacuation time needed rapidly increasing.

In addition, it was also worth noting that when the community density was greater than 120 agents/grid, the percentage of evacuees increased slowly or even stopped growing for a period of time (as shown in Figure 6). For example, when the community density was 160 agents/grid, the evacuation ratio slowly increased after reaching 80%. Besides, the percentage of evacuated agents did not increase for a period time. The reason for this phenomenon may be that a number of resident agents farther away from the shelter gather on the road intersections, which made the road intersections crowded and the moving speed of resident agents decreased. These results implied that traffic was a very important factor need to be considered in the emergency evacuation of high-density communities.

### 3.2. Impacts of Flood Risk Tolerance Threshold of Resident Agents on the Evacuation Process

The resident agents’ behavioral heterogeneity (i.e., agents’ flood risk tolerance threshold) indicated the different response behavior to the same flood risk condition. As mentioned in Section 2.2.2, the difference of the mean value of the flood risk tolerance threshold mainly influenced the evacuation decision-making timing of resident agents. Therefore, the second scenario was to find out how the mean value of the flood risk tolerance threshold of resident agents affects the evacuation process in communities with different densities. In this section, the difference of the total evacuation times in communities with different densities were compared to investigate this effect.

**Figure 7:**
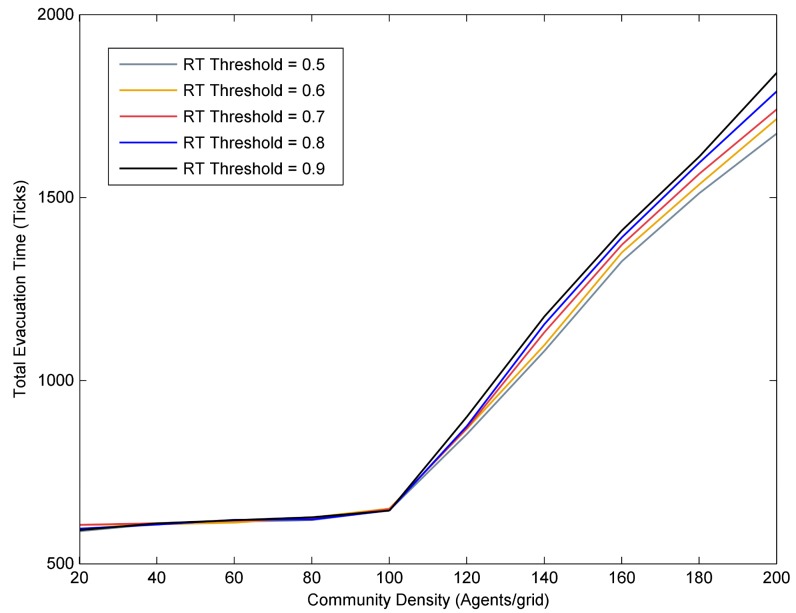
The impacts of residents’ flood risk tolerance threshold on the total evacuation time.

As shown in Figure 7, with regard to the flood risk tolerance threshold, its effects on the evacuation process varied in communities with different densities. In low-density community, such as communities with density less than 120 agents/grid, the difference in the flood risk tolerance threshold had slight impact on the evacuation process. To be specific, at a given community density range from 20 to 100 agents/grid, the total evacuation time fluctuated little no matter how the mean value of the flood risk tolerance threshold of resident agents changed. However, in the high-density communities (i.e., greater than 120 agents/grid), the impact of the flood risk tolerance threshold on the evacuation process can be easily captured. It was clear that as the mean value of the flood risk tolerance threshold increased, more time was needed to evacuate all the resident agents to the shelter, especially in higher density communities, the total evacuation time can be reduced more by reducing the mean value of resident agents’ flood risk tolerance threshold. For example, for two communities with different densities (such as 140 and 200 agents/grid), when the mean value of the flood risk tolerance threshold of resident agents was reduced from 0.9 to 0.5, the total evacuation time of the whole community was respectively reduced by 94 ticks and 166 ticks. These results implied that it was effective to reduce the residents’ flood risk tolerance threshold in high-density communities because it reduced the total evacuation time and improve evacuation efficiency, while it was not effective in low-density communities.

### 3.3. Impacts of Vulnerable Residents on the Evacuation Process

The vulnerability of communities is an important issue that should be considered in the flood risk management of urban communities. As mentioned in Section 2.2.2, the vulnerable residents only evacuate when the community staff or other non-vulnerable residents inform them to leave and move to the shelter together. In general, the movement speed of the vulnerable residents is slower than the non-vulnerable residents. Thus, the number of vulnerable residents in the community would affect the entire community emergency evacuation process because the later evacuation behaviors and the slower movement speed of the vulnerable resident agents.

**Figure 8:**
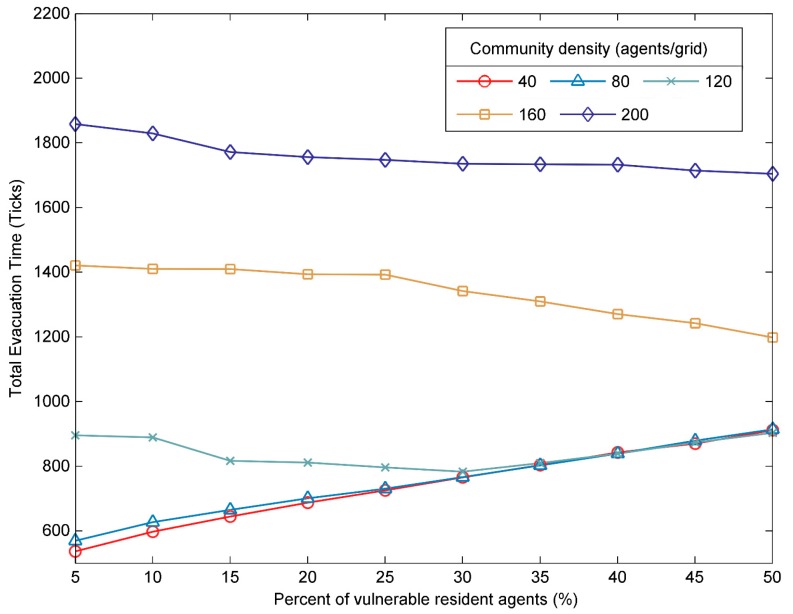
The impacts of the percent of vulnerable resident agents on the total evacuation time.

In this section, the relationship between the proportion of the vulnerable resident agents and the total evacuation time was studied to investigate how the vulnerable residents affects the evacuation process in communities with different densities. Communities were divided into three types, namely, low-density, medium-density and high-density communities. There were less than 120 agents/grid in low-density communities and more than 120 agents/grid in high-density communities. As shown in Figure 8, with the increase of the proportion of vulnerable resident agents, the total evacuation time showed different trends in the three types of communities. To be specific, (1) in low-density communities, the time required to evacuate all agents increased significantly as the proportion of vulnerable resident agents increased, and the result was similar to the previous study [26]; (2) in the medium-density communities, the total evacuation time firstly showed a slight trend of decline when the proportion of vulnerable resident agents increased from 5% to 30%, and then gradually increased when the proportion of vulnerable resident agents continued to increase; (3) in the high-density communities, the increase in the percentage of vulnerable resident agents resulted in a slight decrease in total evacuation time, which was significantly different from the other two types of communities. These results demonstrated that the proportion of vulnerable resident agents is an important factor in community evacuation.

**Figure 9:**
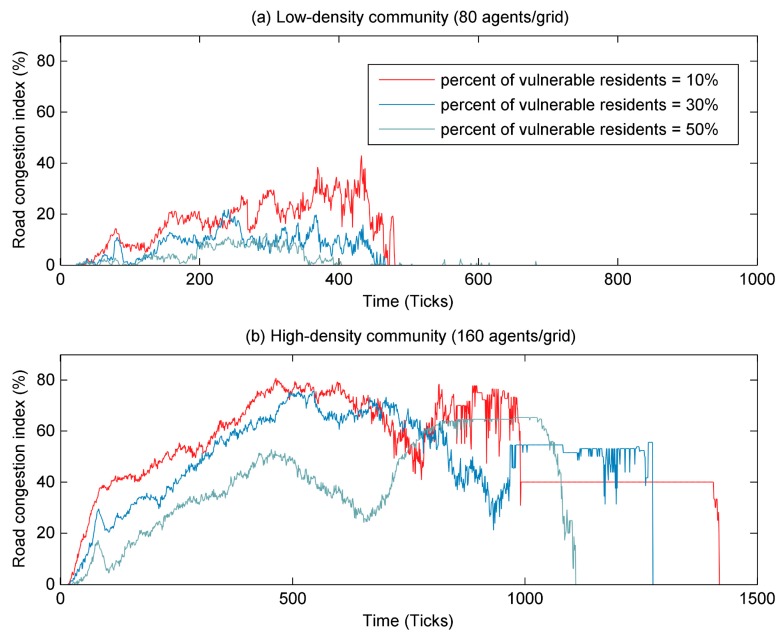
The changes in road congestion index with time in different communities. The percent of vulnerable resident agents was set as 10%, 30% and 50%. (**a**) The road congestion index in a low-density community (80 agents/grid); (**b**) The road congestion index in a high-density community (160 agents/grid).

Then, the road congestion index was used to explore why the proportion of vulnerable resident agents had different effects on the evacuation process in three types of communities. As shown in Figure 9a, it can be found that in low-density communities, the congestion degree and the duration of congestion were relatively short (e.g., RCI less than 40%), and the increase in the proportion of vulnerable resident agents did not significantly affect the degree of road congestion. This was because a low number of resident agents did not significantly contribute to road congestion. Although the road congestion index in low-density communities was low, the increase in density can also lead to an increase in total evacuation time (as shown in Figure 8). This was mainly due to the slower movement speed of vulnerable resident agents, which required more time to evacuate. In addition, it can also be seen from Figure 9b that in high-density communities, the degree of road congestion remained at a high level, with RCI greater than 50% most of the time. However, an appropriate increase in the proportion of vulnerable resident agents could reduce the degree of road congestion, thus accelerating the evacuation of residents and reducing the evacuation time. For example, from 0 to 650 ticks, the higher the proportion of vulnerable resident agents, the lower the road congestion index. This was because vulnerable residents were evacuated later than non-vulnerable residents, so the evacuation process could be carried out in batches. Especially when the proportion of vulnerable resident agents was large, it can avoid a large number of resident agents pouring into the road at the same time, thus reducing the total evacuation time.

These results implied that in low-density communities, the total evacuation time was mainly affected by the movement speed of vulnerable residents, while in high-density communities, the road congestion caused by a large number of residents was the main factor. With regard to the medium-density communities, as the proportion of vulnerable resident agents increased, total evacuation time was first mainly affected by the movement speed of vulnerable resident agents and then affected by the road congestion. Therefore, in vulnerable communities with different densities, government decision-makers need to formulate different evacuation policies according to the evacuation characteristics of the communities.

### 3.4. Impacts of Community Mutual Aid Mechanism on the Evacuation Process

**Figure 10:**
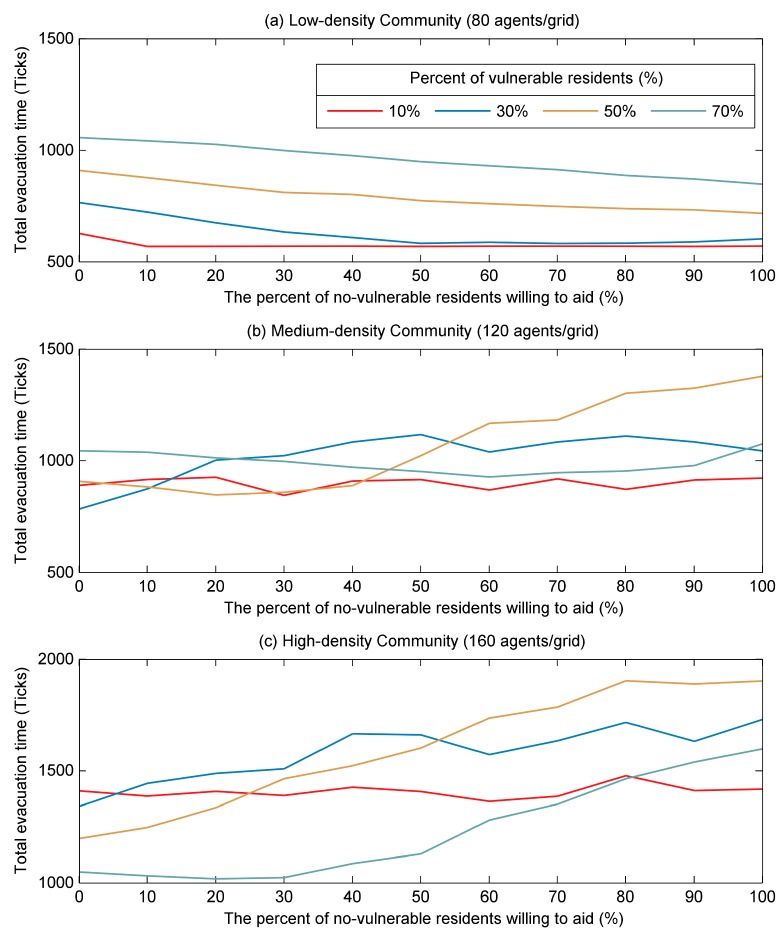
The impacts of the percent of non-vulnerable residents willing to aid on the total evacuation time. (**a**–**c**) The community density is 80, 120, and 160 agents/grid, respectively representing low-density, medium-density and high-density community. The percent of vulnerable residents (v) was set as 10%, 30%, 50%, and 70%.

Mutual aid behavior between neighbors is a common way to respond to the flood disaster events. However, whether this kind of behavior can significantly reduce evacuation time and improve evacuation efficiency has not been widely concerned and is worth studying. In order to investigate how the mutual aid mechanism affects the evacuation process of different communities, in this study, the percent of non-vulnerable resident agents willing to aid was used to indicate the mutual aid mechanism between neighbors. Besides, the community density and the percent of vulnerable resident agents were considered together to represent different types of communities. Similar to the Section 3.3, communities were divided into three categories: low-density, medium-density, and high-density. The results of the total evacuation time in different types of communities under the influence of mutual aid mechanism were shown in Figure 10.

In the low-density community, as shown in Figure 10a, it can be found that the mutual aid behavior between neighbors was effective and can reduce the total evacuation time significantly. When the problem of community vulnerability was serious (e.g., the proportion of vulnerable resident agents was 50% and 70%), more aid from non-vulnerable residents can significantly reduce the total evacuation time. To be specific, the total evacuation time decreased by 20% when the proportion of non-vulnerable residents willing to aid increased from 0% to 100%. However, when the proportion of vulnerable residents was 10% and 30%, the total evacuation time firstly decreased and then remained almost unchanged after the percent of non-vulnerable residents willing to aid increased to a certain proportion. This was because when the number of non-vulnerable residents willing to aid was equal to the number of vulnerable resident agents, all the non-vulnerable residents had been aided to go to the shelter. Therefore, continuing to increase the number of non-vulnerable residents willing to aid would no longer affect the total evacuation time.

With regard to the evacuation in the medium-density community, as shown in Figure 10b, the results indicated that the mutual aid mechanism between neighbors had little effect on the total evacuation time. For example, for a medium-density community with 10% or 70% vulnerable residents, the total evacuation time barely changed regardless of how many non-vulnerable residents were willing to aid. As for the community with 50% vulnerable residents, the time needed to evacuate all residents began to increase significantly after the percent of non-vulnerable residents willing to help reached to 40%. These results implied that the mutual aid mechanism between neighbors slightly affect the evacuation process in medium-density community, especially when the percent of the vulnerable residents was low or high (e.g., 10% and 70%). In other words, managers of medium-density communities should not promote a high percent of mutual aid behaviors.

In the emergency evacuation of high-density communities, especially those with a high proportion of vulnerable residents, the mutual aid mechanism was ineffective and actually increased the total evacuation time (as shown in Figure 10c). This result was completely contrary to the result of low-density communities. Specifically, for communities with 30% or more vulnerable residents, the total evacuation time increased significantly as more non-vulnerable residents were willing to aid. For example, in communities with 50% vulnerable residents, when the percentage of non-vulnerable residents willing to aid increased from 0 to 50%, the total evacuation time increased by 33.6%, and when the aid ratio increased to 100%, the evacuation time significantly increased by up to 58.7% percent. This may be because in high-density communities, the greater number of non-vulnerable residents help vulnerable residents to go to the shelter together, the more likely that a large number of residents would pour into the road at a certain moment, which would cause traffic congestion and increase evacuation time. The results suggested that the more non-vulnerable residents offered aid in high-density community, the less efficient evacuation because the total evacuation time increased significantly.

## 4. Conclusions

In this study, an agent-based modelling framework that incorporated the resident psychological model and transportation network model was conducted to explore the influence of community properties, resident’s psychological attributes and mutual aid mechanism on the flood evacuation processes. Specifically, four main factors: community density, flood risk tolerance threshold of residents, percent of vulnerable residents and mutual aid mechanism were considered together to investigate how they interplay with each other to affect agents’ flood evacuation process. Indicators such as total evacuation time, mean evacuation time, percent of evacuated agents and road congestion index were used to evaluate the evacuation efficiency of the whole community. The advantages of this study were mainly in three aspects. Firstly, this study considered residents’ self-rescue and community staff’s assistance at the same time which is more in line with the reality of community evacuation. Secondly, different from previous evacuation studies, this study focused on vulnerable communities, and the results can identify the main difficulties in evacuation in different types of vulnerable communities. Thirdly, this study highlighted the mutual aid mechanism among residents in the evacuation process, which were usually neglected in previous evacuation studies. The influence of mutual aid mechanism in different communities were identified and the results can provide community managers with information on how to deal with mutual aid behaviors in different types of communities.

The key findings from the simulation results under different scenarios were: (1) The community density was found to be negatively correlated with flood evacuation efficiency. Achieving a high level of evacuation ratio was much more difficult in high-density community than in low-density community due to the marginal time needed rapidly increasing. That was because evacuations from high-density communities can easily cause road congestion. (2) Lower flood risk tolerance threshold can help residents respond to flood disasters timely, but this effect was different in communities with different densities. In high-density communities, appropriately reducing the value of flood risk tolerance threshold of residents can obviously reduce the total evacuation time and improve evacuation efficiency, however, it was not effective in low-density communities. (3) The proportion of vulnerable resident agents had opposite effects in different types of communities. With the increase of the proportion of vulnerable resident agents, the total evacuation time increased significantly in low-density communities, decreased first and then increased in medium-density communities, and decreased in high-density communities. (4) Mutual aid mechanism can reduce evacuation time in low-density communities, and the effect was more pronounced with a higher proportion of vulnerable resident agents in the community. However, in high-density communities, the mutual aid mechanism would actually increase the evacuation time. Besides, the higher the proportion of vulnerable resident agents, the more the evacuation time would increase. In the medium-density community, the percent of non-vulnerable resident willing to aid had a negative relationship with total evacuation time, but it was not obvious.

Such information can help urban community managers and decision-makers increase disaster evacuation efficiency by developing different evacuation strategies according to the specific characteristics of different communities. For example, managers must pay special attention to the impact of community density, especially evacuation in high-density communities. Appropriately lowering the residents’ flood risk tolerance threshold could prompt residents to make evacuation decisions earlier. The change of the proportion of vulnerable residents in low and medium-density communities can easily affect the evacuation efficiency. Community managers can adopt various means of transportation to improve the evacuation speed and encourage mutual aid behaviors between residents to reduce the evacuation time. While evacuation in the high-density community, the traffic congestion is the main challenge. Community managers should avoid road congestion caused by the mutual aid behaviors among a large number of people, and should arrange for evacuations in batches when road capacity is inadequate.

But, the limitation of the study should also be taken into consideration. Firstly, in order to make the agent-based model uncomplicated, feasible and operable, not all the factors affecting the residents’ evacuation process were considered in this study. For example, the families or friends were assumed to evacuate at the same time in this study, but in reality, residents’ behaviors of seeking relatives and friends during the evacuation are common. These searching behaviors are more complex and can affect the simulation results, which will be considered in future studies. Secondly, the agent-based model was conducted in a synthetic community and the usability of the model was verified by comparing the simulation results with previous studies and empirical data. However, due to the difficulty in obtaining more detailed evacuation data, it is difficult to further verify the simulation results of the entire evacuation process. Thirdly, the more parameters in ABM model, the more likely random results will appear. In this study, the model was running 500 times and calculated the truncated mean value to avoid randomness. However, it would be more time-consuming and labor consuming to increase the number of runs in order to get more stable model results. Even so, some interesting results were found and can be used to optimize flood risk management in urban community.

Future work will use the empirical data to measure the various behavioral parameters, modify and improve the mutual aid mechanism based on community interviews and survey results, use realistic communities and transportation networks as the research areas, consider the dynamic impact of flood events, and combine the spatial-temporal uncertainty of multi-stage flood warnings.

## Figures and Tables

**Table 1:** List of resident agents’ attributes.

Category	Variables	Description
Physical	i	Unique identification number of each agent
	ES	Evacuation status of agents
	GL	Geographical location of agents in the community
	CGN	Community grid number where agents live
	V_max_	Agent’s max movement speed during the evacuation
	CAT	Agent’s categories (non-vulnerable resident and vulnerable resident)
Psychological	RT	Agent’s flood risk tolerance threshold
	FRP	Agent’s flood risk perception

**Table 2:** Description of different categories of resident agent.

Category	Speed	Interaction Rules	Representative
Non-vulnerable resident agent	fast	- evacuate by themselves when its flood risk perception greater than the its flood risk tolerance threshold	Young adults
Vulnerable resident agent	slow	- evacuate when community staff find them and ask them to leave - evacuate when other non-vulnerable resident agent helps them to evacuate	Children, elderly, people with mobility problems

**Table 3:** The Values of Model Parameters.

Parameters	FRW	$\alpha_{j}$	$\beta_{j}$	$\omega_{j}$
Values	1	0.5	0.5	0.5(0.1) ^a^

^a^$x_{1}(x_{2})$ indicates the value of the parameter is sampled from a normal distribution with a mean of $x_{1}$ and standard-deviation of $x_{2}$.

**Table 4:** Parameters Design for Four Scenarios.

Scenario	density	risk_tolerance	vulnerable_pct	aid_stat	help_pct
Scenario 1	10:10:200 ^a^	0.7(0.05) ^b^	0.1	false	0.0
Scenario 2	10:20:200	0.5:0.1:0.9	0.1	false	0.0
Scenario 3	10:20:200	0.7(0.05)	0.1:0.1:0.5	false	0.0
Scenario 4	10:20:200	0.7(0.05)	0.1:0.1:0.5	true	0.1:0.1:1

^a^$x_{1}:d:x_{2}$ denotes a numeric vector from $x_{1}$ to $x_{2}$ with increment of d. For example, in scenario 2, the mean value of flood risk tolerance threshold 0.5:0.1:0.9 means vector [0.5 0.6 0.7 0.8 0.9]. ^b^ $x_{1}(x_{2})$ indicates the value of the parameter is sampled from a normal distribution with a mean of $x_{1}$ and standard-deviation of $x_{2}$.
